# Optical sensing nanostructures for porous silicon rugate filters

**DOI:** 10.1186/1556-276X-7-79

**Published:** 2012-01-17

**Authors:** Sha Li, Dehong Hu, Jianfeng Huang, Lintao Cai

**Affiliations:** 1 CAS Key Lab of Health Informatics, Shenzhen Key Laboratory of Cancer Nanotechnology, Institute of Biomedical and Health Engineering, Shenzhen Institutes of Advanced Technology, Chinese Academy of Sciences, Xueyuan Avenue 1068, Shenzhen University Town, Shenzhen, 518055, People's Republic of China

## Abstract

Porous silicon rugate filters [PSRFs] and combination PSRFs [C-PSRFs] are emerging as interesting sensing materials due to their specific nanostructures and superior optical properties. In this work, we present a systematic study of the PSRF fabrication and its nanostructure/optical characterization. Various PSRF chips were produced with resonance peaks that are adjustable from visible region to near-infrared region by simply increasing the periods of sine currents in a programmed electrochemical etching method. A regression analysis revealed a perfect linear correlation between the resonant peak wavelength and the period of etching current. By coupling the sine currents with several different periods, C-PSRFs were produced with defined multiple resonance peaks located at desired positions. A scanning electron microscope and a microfiber spectrophotometer were employed to analyze their physical structure and feature spectra, respectively. The sensing properties of C-PSRFs were investigated in an ethanol vapor, where the red shifts of the C-PSRF peaks had a good linear relationship with a certain concentration of ethanol vapor. As the concentration increased, the slope of the regression line also increased. The C-PSRF sensors indicated the high sensitivity, quick response, perfect durability, reproducibility, and versatility in other organic gas sensing.

## Background

Porous silicon [PSi], a material with unique structural and optical properties, can be prepared by anodic etching of silicon in ethanolic hydrofluoric acid solution [1-3]. By changing the current density, the porosity of PSi that decides the refractive index can be tailored in a wide range, and thus, it is able to obtain many types of PSi optical structures such as porous silicon microcavities and porous silicon rugate filters [PSRFs]. Among them, PSRFs are a class of multilayered photonic crystal with a sinusoidal refractive index distribution that is normal to the surface. Light incident on the surface of a rugate filter will be reflected in a narrow spectral range, and spectral position is dependent on the refractive index of the material [4]. PSRFs were introduced and further improved afterwards by different groups [5-9]. For example, by placing the electrode in a different way, a porous silicon band filter gradient that displayed rainbow colors on its surface was prepared [6], and by employing an amplitude-modulated sinusoidal refractive index apodized with a Gaussian function, the optical property was greatly improved as a stop band at a wavelength of 850 nm yet with the full-width at half maximum [FWHM] being only 5 nm [7,8]. Notably, the location of its peak was found to be determined by the period of the waveform used in the preparation [9].

Recently, combination PSRFs [C-PSRFs] have been developed. One kind of C-PSRF was designed with two peaks in their reflectance spectra and combined with two multilayered mirrors, among which the hydrophobic one was at the top and the hydrophilic one, at the bottom [10,11]. These C-PSRFs have been used to manipulate the movement of liquid droplets [10] and local heating [11]. Another kind of C-PSRF was constituted by combined multilayered mirrors and generated by coupling *n *sine waves with different frequencies used for PSRF preparation [12]. By contrast, these C-PSRFs were applied for biomolecular screening [5] or encoded microcarriers [12]. However, the nanostructure and optical characterization have not been studied systematically.

Herein, we reported a systematic study on the fabrication and characterization of PSRFs and C-PSRFs, especially on the relationships of position, FWHM, and intensity of their optical resonance peaks with the period of sinusoidal current density applied in the synthesis. Also, a scanning electron microscope and a microfiber spectrophotometer were employed to analyze their physical nanostructure and feature spectra, respectively. The novel C-PSRF with three synchronous peaks was used to detect the response to ethanol vapor, and the red shifts of the C-PSRF peaks had a good linear relationship with a certain concentration of ethanol vapor. As the concentration increased, the slope of the regression line also increased. The C-PSRF sensors indicated high sensitivity, quick response, perfect durability, and reproducibility.

## Methods

### Apparatus

Thickness and configuration measurements of the resulting PSRFs were carried out with a field-emission scanning electron microscope [FESEM] (S4700, Hitachi High-Tech, Minato-ku, Tokyo, Japan). The reflectance spectra (200 to 1,100 nm) of the PSRFs were collected by a fiber optic spectrometer (AvaLight-DHS, Avantes BV, Apeldoorn, The Netherlands) with a halogen lamp as light source. The resolution of the spectrometer is 0.8 nm.

### Reagents

The highly doped p-type Si wafers (boron-doped, 0.002 to 0.004 Ω cm resistivity) were obtained from Silicon Valley Microelectronics, Inc. (Santa Clara, CA, USA). Hydrofluoric acid [HF] was obtained from Chemical Reagent Company, Ltd. of Dongguan City, Guangdong, China. All other chemicals used in this study were of analytical reagent grade and used without further purification. A JL-RO100 Milli-pore-Q Plus water (Millipore Co., Billerica, MA, USA) purifier supplied deionized water with a resistivity of 18.25 MΩ cm.

### PSRF preparation

**Figure 1:**
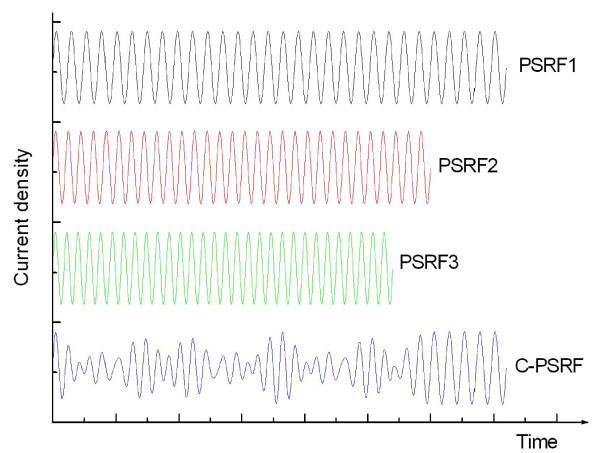
**The current densities for PSRFs and the coupled density for C-PSRFs**.

PSRFs were prepared by electrochemical anodization on highly doped p-type Si wafers (boron-doped, 0.002 to 0.004 Ω cm resistivity). Immediately before anodization, the substrates were cleaned in propanol in an ultrasonic bath and rinsed in deionized water. Anodization was performed under standard galvanostatic conditions in a 3:1 (*v*/*v*) solution of 49% aqueous HF and ethanol. A Teflon etch cell that exposed 0.46 cm^2 ^of the Si wafer was employed. A platinum mesh electrode was immersed into the electrolyte as a counter electrode, and silicon was anodized using a computer-controlled current source (2400 SourceMeter, Kiethley Instruments Inc., Cleveland, OH, USA). The current density was modulated with a sinusoidal waveform (Figure 1) varying from 10 to 50 mA cm^-2 ^and cycled 30 times. Such a wide range of the current density was indispensable to achieve a porosity modulation in the structure. The periods for these sine waves were between 2.4 to 6.9 s. After formation, the samples were rinsed with pure ethanol and were dried with nitrogen gas.

### C-PSRF preparation

Three sine waves with 30 periods and different frequencies (e.g., PSRF1, PSRF2, and PSRF3; Figure 1) were coupled to generate a combined waveform (Figure 1) that was then converted into a current-time waveform by the computer-controlled current source (2400 SourceMeter, Kiethley Instruments Inc., Cleveland, OH, USA). The converted waveform was then applied to etch a porosity-depth profile in the Si wafer, yielding the C-PSRFs [13]. Other conditions were kept the same as described in the PSRF preparation.

Prior to anodization, all the silicon wafers were dipped in 5 wt.% HF solution to remove the native oxides. After anodization, all the samples were rinsed with ethanol and then dried under a gentle stream of nitrogen gas.

### Ethanol sensing

For ethanol monitoring experiments, the PSRF wafers were placed in a sealed steel chamber with a window that was covered with a quartz glass, in which the detecting vapors were transported at room temperature. The dry ethanol vapors were produced by the bubbling of nitrogen with a flow rate of 100 sccm into an ethanol aqueous solution with different concentrations and dried by passing through a pipe filled with anhydrous Na_2_CO_3_.

## Results and discussion

**Figure 2:**
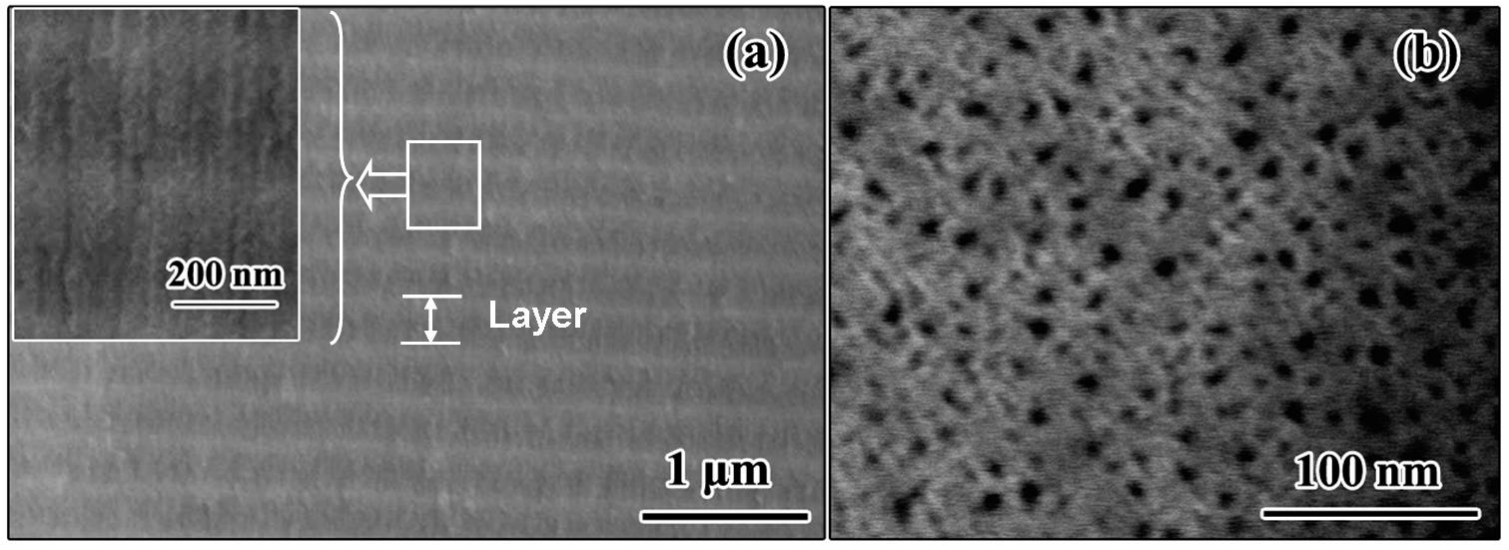
**Cross-sectional view (a) and top view (b) of PSRF wafer FESEM images**. The inset is a magnified cross-sectional view. (PSRF was prepared with a sinusoidal waveform ranging between 10 and 50 mA cm^-2 ^with a period of 6.9 s for 30 cycles in a 3:1 (*v*/*v*) solution of 49% aqueous hydrofluoric acid and ethanol.)

The PSRF structure was observed in various aspects using a FESEM. As can be seen from Figure 2a, the PSRF showed a sinusoidal-varying porosity gradient in the direction perpendicular to the plane of the filter, where the higher porosity film by high-current etching was in the dark zone, whereas the lower porosity film by low-current etching was located in the bright zone. The whole thickness for the stack layer of each sinusoidal period was about 285 nm on average. A magnified cross-sectional view (inset) revealed that the porous structure was made up of nanopores, where the inside walls of the nanopores were rather rough. Figure 2b showed the top view SEM image of the PSRF, where the pore sizes were 2 to 10 nm.

**Figure 3:**
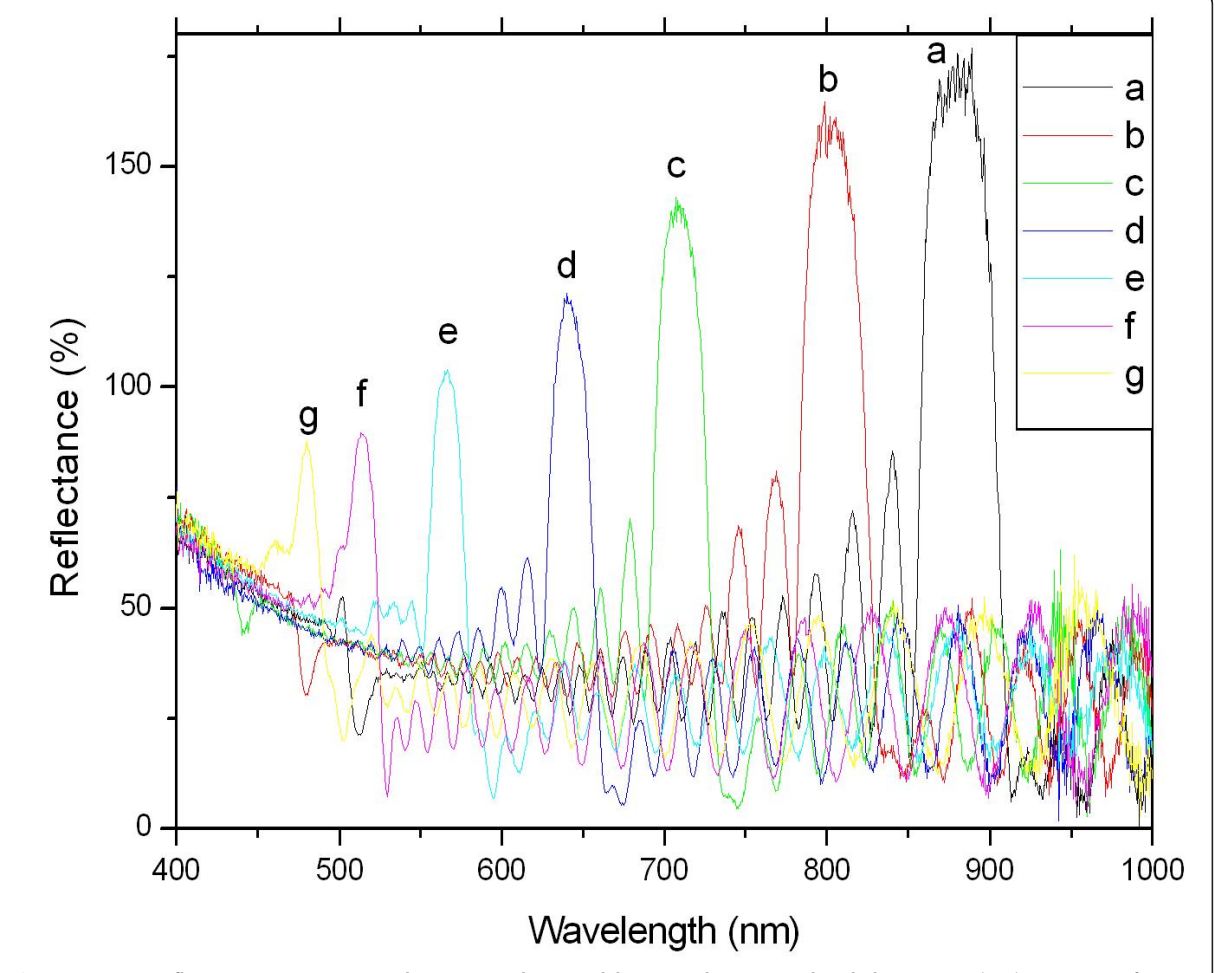
**PSRFs' reflectance spectra prepared using varied sinusoidal current densities and cycled 30 times**. The densities were from 10 to 50 mA cm^-2^, and the different periods for the curves (*a *to *g*) were 5.52, 4.83, 4.14, 3.893, 3.105, 2.76, and 2.415 s, respectively.

**Figure 4:**
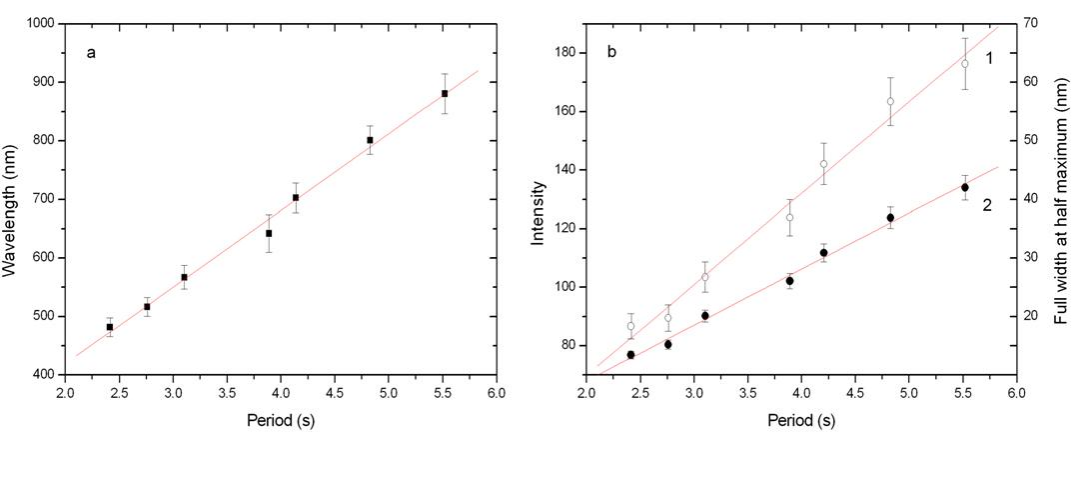
**Linear relationships**. (**a**) Linear relationships between the wavelength and the period (filled square). (**b**) Linear relationships between (1) the intensity and the period (empty circle) and (2) the FWHM and the period (filled circle).

Figure 3 showed the reflectance spectra of a series of PSRFs produced with sinusoidal current densities of different periods. It was clear that there was a resonance peak in each feature spectrum. As the current periods increased, the resonance peak shifted from the visible region to the near-infrared region, and the FWHM also increased. A regression analysis revealed a perfect linear correlation between the resonant peak wavelength (*λ*, nanometer) and the etching time (*T*, seconds) (*λ *= 161.961 + 129.659 *T, R *= 0.996; Figure 4a).

As the porosity could be controlled by the etching current density, with the pore dimensions in these structures too small to effectively scatter light and each porous layer treated as a single medium with a single refractive index value, the resulting linear relation between the peak wavelength (*λ*) and the period of the sinusoidal current (*T*) described above could be explained in Equation 1 as follows:

(1) mλ = 2nL,

where *m *was the spectral order of the fringe at wavelength *λ, n *was the refractive index, *L *was the thickness of the film, and *nL *was the optical thickness [14-16].

It was obvious that the PSRFs with peaks ranging from short to long wavelengths could be obtained by increasing the thickness of each layer, which was controlled by the duration of the etch cycle [5].

In the meantime, it can be seen that the intensity and FWHM of those peaks increased along with the peak wavelength. A linear relationship of the peak intensity (*I*) and the period (*T*) was obtained as *I *= -10.169 + 9.570 *T *(s) with a linear correlation coefficient of *R *= 0.997 (number 1 in Figure 4b), and the relationship between FWHM and the period was FWHM = 7.370 + 31.188 *T *(s) with a linear correlation coefficient of *R *= 0.993 (number 2 in Figure 4b). The linear strengthening intensity was mainly caused by the increasing photon number that was the result of the linear increasing thickness of the optical structure layer and the easier photonic diffraction when the peak wavelength increased. As to the same reason why the FWHM linearly broadened, it could be derived from Equation 2:

(2) $$\lambda =\frac{hc}{E}=\frac{6.63\times 10^{-34}\times 3.00\times 10^{8}\;}{W\times 1.6\times 10^{-19}}\left(\text{m}\right)=\frac{1243.125}{W}\left(\text{nm}\right),$$

where *λ *was the wavelength of peak in the spectrum, *h *was Planck's constant, *c *was the speed of light, and energy (*E*, joules) and work (*W*, electron volt) were photon energies. As can be seen, the wavelength (***λ***) red shifts resulted in the lower photon energy (*W*).

**Figure 5:**
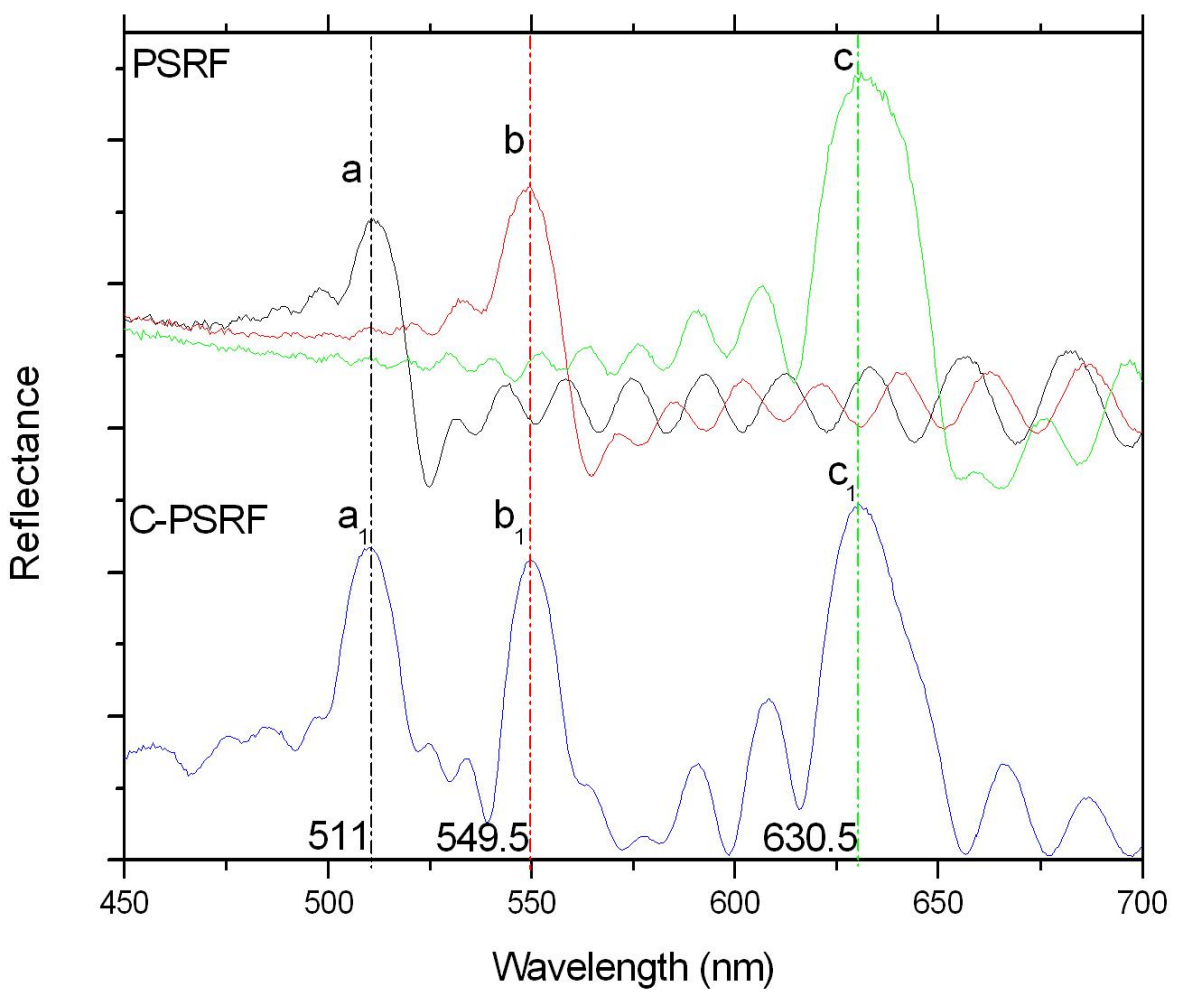
**Reflectance spectra of PSRFs and C-PSRFs**. PSRFs' reflectance spectra (upper curve) etched with single periods of (*a*) 3.105, (*b*) 3.45, and (*c*) 4.14 s, respectively, and reflectance spectrum of C-PSRFs (lower curve) with three peaks etched with the coupled current density of three single periods of (*a_1_*) 3.105, (*b_1_*) 3.45, and (*c_1_*) 4.14 s. In both cases, the sinusoidal current varied between 10 and 50 mA cm^-2 ^and was cycled 30 times.

Based on the discussion above, C-PSRFs were designed using three sine waves with periods of 3.105, 3.45, and 4.14 s, where the combined current were coupled to generate a combination waveform by the computer-controlled current source, etching a porosity-depth profile into the Si wafer. By comparing the peak positions of the C-PSRFs (i.e., 510.7, 549.9, and 630.5 nm) and PSRFs (i.e., 511.4, 549.4, and 630.5 nm), it indicated that the peak positions of C-PSRFs were exactly kept in the same peak positions as in the single PSRFs with the difference of less than 1 nm (Figure 5). All the sinusoidal current densities used in this work were of the same variation from 10 to 50 mA cm^-2 ^for 30 cycles. Moreover, we have already produced C-PSRFs with two resonance peaks by the same process, so C-PSRFs with specific peak numbers and peak positions could be controllably produced by modulating the numbers and periods of the combined current by single sine waves that were coupled. In addition, it could be seen that the peak intensity varied, which could be attributed to the interaction of the three coupled sine waves and the optical structures.

**Figure 6:**
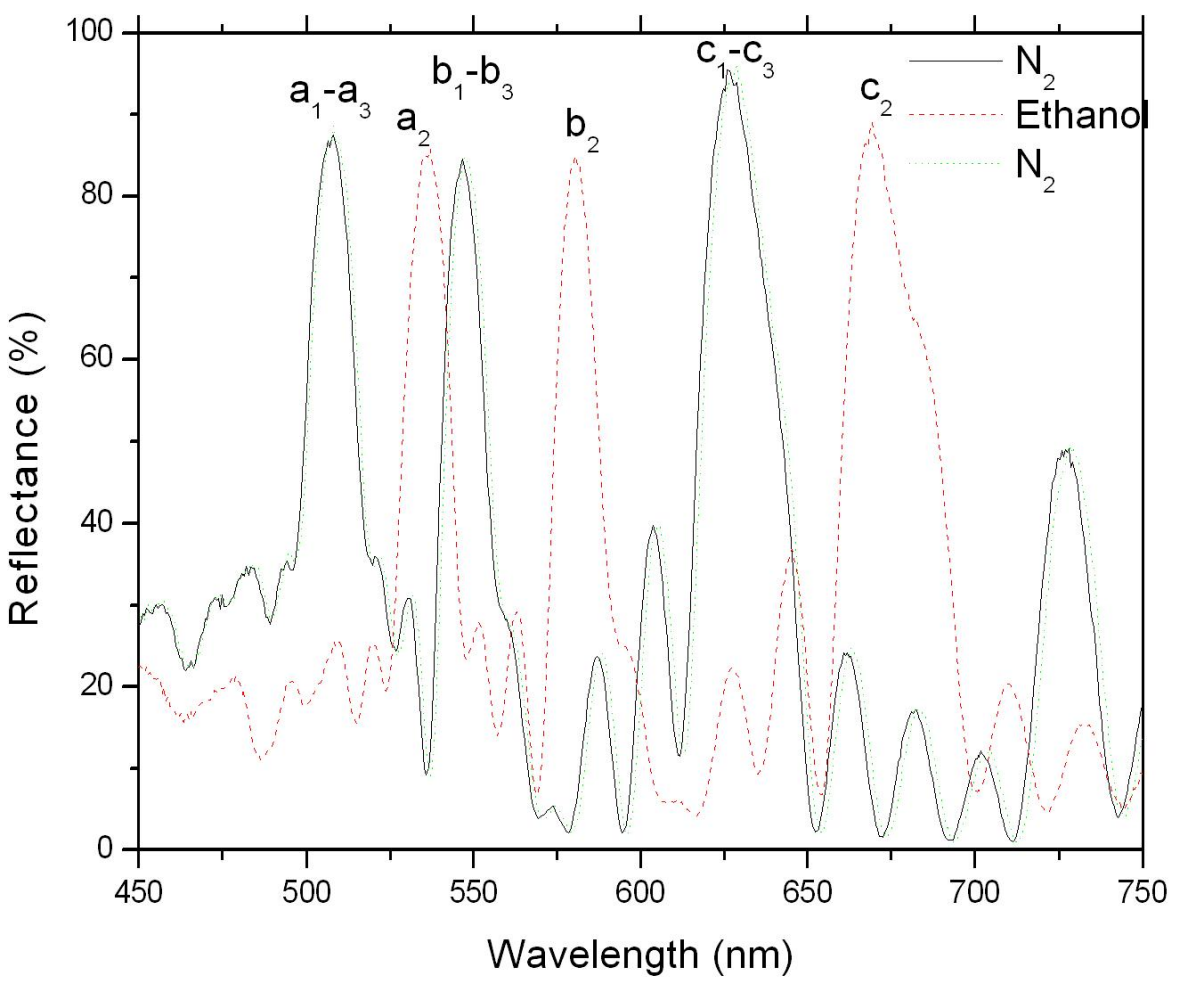
**Reflectance spectra of C-PSRF**. C-PSRF when exposed to N_2 _(*a_1_ , b_1_*, and *c_1_*), gas mixture of N_2 _and ethanol vapor (*a_2_ , b_2_*, and *c_2_*), and N_2 _(*a_3_ , b_3_*, and *c_3_*).

C-PSRF was a type of promising sensing material for ethanol optical sensors. As shown in Figure 6, upon the exposure of ethanol vapor, the C-PSRF showed dramatic red shifts in its peaks. Specifically, the peaks at the positions of a_1_, b_1_, and c_1 _shifted to the positions of a_2_, b_2_, and c_2_, respectively, and the amounts of the red shifts for a_1_, b_1_, and c_1 _were 26.4, 31.1, and 40.2 nm, respectively. Obviously, under the same conditions, the red shifts for three peaks were different. A perfect linear relationship between the amounts of red shifts (Rs, nanometer) and the wavelengths of the original peaks (*λ*, nanometer) could be obtained as Rs = -30.968 + 0.109 *λ *with a correlation coefficient of *R *= 1.000.

When the ethanol vapors were desorbed through exposure of N_2_, the peaks would quickly return to their exact initial positions (see a_3_, b_3_, and c_3 _in Figure 6). This process was completely reversible and repeatable even after several cycles of exposure. The same value for each measurement within the experiment error has been obtained from the process that has been repeated three times. The response time to ethanol vapors and recovery time from N_2 _were as rapid as within 5 s.

**Figure 7:**
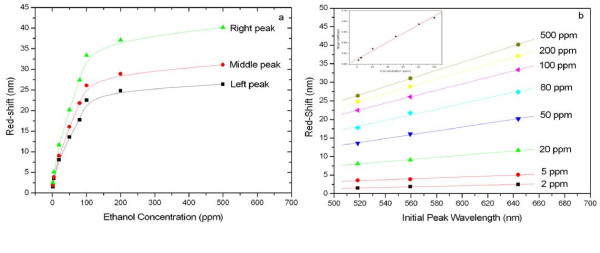
**Red shifts for the three peaks and their corresponding linear correlations**. (**a**) Red shifts for the three peaks of C-PSRFs when exposed to ethanol vapor with different concentrations. (**b**) The linear correlations between the red shifts of the three peaks and the peaks' initial positions when exposed to a certain concentration of ethanol vapor. The inset is the linear correlation between the slope coefficient and the concentration of ethanol.

To further study the sensing properties of C-PSRFs, a gas mixture of N_2 _with ethanol vapor at different concentrations (Figure 7a) was exposed to the chip. Figure 7a showed the corresponding red shifts for the three peaks as a function of the ethanol concentration. At a low concentration (< 100 ppm), the red shifts (Rs, nanometer) gave a good linear relationship with the concentration (*C*, parts per million). The regression equations for three peaks, i.e., left, middle, and right peaks, were Rs_1 _= 2.623 + 0.199 *C*_1 _(correlation *R*_1 _= 0.991), Rs_2 _= 2.933 + 0.239 *C*_2 _(correlation *R*_2 _= 0.993), and Rs_3 _= 3.794 + 0.302 *C*_3 _(correlation *R*_3 _= 0.994), respectively. Calculated from the slopes of the equations, obtained were the sensitivities of the three peaks as 0.200, 0.239, and 0.302 nm ppm^-1^. Notably, compared with the two left peaks, the right peak that was situated at a longer wavelength displayed the highest sensitivity. Figure 7b illustrated the red shift of each peak as a function of the peak's initial position when ethanol vapor with different concentrations was exposed to the C-PSRF chip. As can be seen, the shifts for each peak show a good linear relationship with the peaks' initial positions when a certain concentration of ethanol was exposed, and the slope for the regression line increases with the concentration. Specifically, the slope coefficients for these lines were calculated from the bottom to the top as 0.0079, 0.012, 0.0290, 0.0521, 0.0751, 0.087, 0.098, and 0.1098. Hence, another linear equation of the slope coefficient (*K*) as a function of their corresponding concentration (*C*, parts per million) could be obtained as *K *= 0.0095 + 0.0008 *C *with a correlation of *R *= 0.996 (see the inset of Figure 7b). Therefore, based on the results of the standard curves mentioned above, whatever red shift of the peaks (i.e., left, middle, or right) or slope coefficient of the three peaks was measured, it gave way for the calculation of the corresponding concentration of ethanol employed in the sensing experiments. The peak located at a shorter wavelength held a small FWHM, while the peak situated at a longer wavelength provided a larger red shift in the sensing process. Thus, the C-PSRF sensors preformed with good selectivity, high sensitivity, and multiplex detection together to be great and efficient, and thus promising in sensing applications.

While these values reflect the sensitivity to a given volume (or mass) of the analyte, it is important to point out that the slope coefficient for a given analyte is profoundly influenced by the adsorption and microcapillary condensation processes operative in these PSRF sensors. The high surface area and the existence of a large volume fraction of micropores both contribute in concentrating the analyte vapors in the sensor. Consequently, the slope coefficient is expected to be substantially reduced, in particular, for molecules that have high sticking probabilities and low vapor pressures [13].

## Conclusions

In summary, we have presented a systematic study on the preparation of PSRFs with resonant peaks varying from the visible region to the near-infrared region. Those PSRFs were electrochemically produced in a program-controlled current etching by systematically tuning the periods of the sine currents. The PSRF resonant peak wavelength was observed as a perfect linear correlation with the period of etching current. Moreover, C-PSRFs with several peaks in the feature spectra were produced using a combination sine current that was coupled with several single sine waves with different frequencies. The relationships between the wavelength of the resonant peak, FWHM, peak intensity, and the periods were revealed. Based on the C-PSRFs' unique nanostructure and multiplex peak detection, the sensing properties of C-PSRFs were explored in the ethanol vapor. The sensing process of C-PSRFs exhibited high sensitivity, quick response, and reproducible abilities, implying their promising applications in gas sensor and biosensing fields.

## Competing interests

The authors declare that they have no competing interests.

## Authors' contributions

SL carried out the preparation of C-PSRF and drafted the manuscript. DH carried out the ethanol monitoring experiments. JH participated in the ethanol monitoring experiments. LC conceived the study and participated in its design and coordination. All authors read and approved the final manuscript.
